# A Selective G-Quadruplex DNA-Stabilizing Ligand Based on a Cyclic Naphthalene Diimide Derivative

**DOI:** 10.3390/molecules200610963

**Published:** 2015-06-12

**Authors:** Md. Monirul Islam, Satoshi Fujii, Shinobu Sato, Tatsuo Okauchi, Shigeori Takenaka

**Affiliations:** 1 Department of Applied Chemistry, Kyushu Institute of Technology, Kitakyushu, Fukuoka 804-8550, Japan; E-Mails: monirbtge@takenaka.che.kyutech.ac.jp (M.M.I.); shinobu@che.kyutech.ac.jp (S.S.); okauchi@che.kyutech.ac.jp (T.O.); 2 Department of Bioscience and Bioinformatics, Kyushu Institute of Technology, Iizuka, Fukuoka 820-8502, Japan; E-Mail: s.fujii@bio.kyutech.ac.jp

**Keywords:** cyclic naphthalene diimide, G-quartet, human telomeric DNA, promoter region, promoter region, thrombin-binding aptamer

## Abstract

A cyclic naphthalene diimide (cyclic NDI, **1**), carrying a benzene moiety as linker chain, was synthesized and its interaction with G-quadruplex DNAs of a-core and a-coreTT as a human telomeric DNA, c-kit and c-myc as DNA sequence at promoter region, or thrombin-binding aptamer (TBA) studied based on UV-VIS and circular dichroism (CD) spectroscopic techniques, thermal melting temperature measurement, and FRET-melting assay. The circular dichroism spectra showed that **1** induced the formation of different types of G-quadruplex DNA structure. Compound **1** bound to these G-quadruplexes with affinities in the range of 10^6^–10^7^ M^−1^ order and a 2:1 stoichiometry. Compound **1** showed 270-fold higher selectivity for a-core than dsDNA with a preferable a-core binding than a-coreTT, c-kit, c-myc and TBA in the presence of K^+^, which is supported by thermal melting studies. The FRET-melting assay also showed that **1** bound preferentially to human telomeric DNA. Compound **1** showed potent inhibition against telomerase activity with an IC_50_ value of 0.9 μM and preferable binding to G-quadruplexes DNA than our previously published cyclic NDI derivative **3** carrying a benzene moiety as longer linker chain.

## 1. Introduction

Guanine-rich DNA sequences which mainly originate in important regions of the oncogene promoters, telomere, mRNA, ribosomal DNA (rDNA), and thrombin-binding aptamer (TBA) can form G-quadruplex structures [1,2,3]. G-quadruplex DNAs, formed at the telomeric end, can inhibit telomere elongation by telomerase, which are activated in 80%–85% cancer cells, leading to inhibition of telomerase activity [1,4]. G-quadruplex DNAs is known to be formed at promoter regions of the human oncogene that can regulate gene expression at the transcriptional level [3]. DNA aptamers bind to thrombin and inhibit thrombin-catalyzed fibrin, resulting in blood clotting [2]. Thus, guanine-rich sequences have become a very promising target for the development of new anticancer drugs and therapeutic applications, which was attracted a lot of research interest during the last few decades and a few of the resulting compounds have entered preclinical or clinical trials [3].

It has been reported that guanine-rich oligonucleotides could form G-quadruplexes via Hoogsteen hydrogen bonding among four guanine bases arranged in a square planar configuration [4]. G-quadruplex DNA shows diverse structural polymorphism; G-quadruplex DNA can be either parallel or antiparallel, even both conformations (termed hybrid) in some cases [5,6]. This G-quadruplex DNA can fold as a mixture of several different quadruplex forms depending on DNA sequence and extrinsic cation which offers a platform to induce and stabilize the quadruplexes by using small organic molecules [5,6]. This common structural feature poses challenges for the design of ligands with considerable selectivity toward one type of quadruplex over other G-quadruplex structures [6].

Small molecules that stabilize the G-rich single-strand DNA overhang into G-quadruplex can be considered as potential cancer and therapeutic agents [3]. A number of G-quadruplex-binding small molecules have been reported in the last few decades [3,7,8]. Several diverse structural ligands, including telomestatin, oxazole, cationic TMPyP4, anthraquinone, perylene, acridine, and ethidium derivatives have been investigated to evaluate their ability to interact with G-quadruplex DNA and observe their biological functions [3,7,8]. There are a number of macrocyclic structures that have been developed in the last few years as G-quadruplexes DNA binding ligands such as BQQ1, telomestain, oxazole, porphyrin, *etc.* [9], which is a well-established technique to improve the development of G-quadruplex DNA selective drugs. A common feature of these G-quadruplex-binding molecules is the presence of an extended aromatic ring system that allows binding through π-π overlap of terminal G-tetrads [5,9]. Large flat aromatic planar molecules stack on G-tetrads and show high binding selectivity [6]. Non-planar molecules that stack with G-quadruplexes are very rare and bindings are moderate [6]. Some of these G-quadruplex-binders include porphyrin derivatives, oxazoles, perylene derivatives and similar systems [10] that have fused π-ring systems within the molecule and showed various binding selectivity with the G-quadruplexes’ DNA structure. Nowadays, the researchers are focusing on developing G-quadruplex DNA structure-specific and selective binding ligands [6,9] which are important for drug development, cancer research and therapeutic application studies.

**Figure 1:**
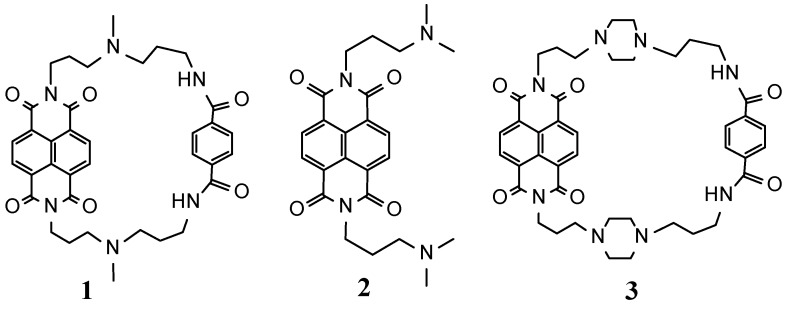
Chemical structures of **1**, **2** and **3** (**3** taken from [10]).

Naphthalene diimides (NDIs) are very potent G-quadruplex-binding ligands with high cellular toxicity, which is able to effectively stabilize the terminal G-quartet of a G-quadruplex by stacking interactions [11,12]. Over the last few years a number of NDI-based compounds have been developed in part by exploiting the available NDI-G-quadruplexes structures [13,14,15,16,17,18,19,20,21]. In our previous studies, we already reported interaction studies of some cyclic NDI derivatives and h-telo 22 G-quadruplex DNA which can inhibit telomerase activity at low concentration [10,22]. In our present work, we synthesized new compound **1** by cyclization with the linker chain of a tertiary amino group and amide group through benzene to compare the binding selectivity with our previously reported compound **3** [10] (Figure 1). Compound **1** is expected to show reduced binding to dsDNA and increased binding affinity for G-quadruplexes DNA because of its shorter linker substituents. We have also sought to compare the binding selectivity among the various structures of G-quadruplex DNA. We have characterized the binding selectivity and stability of **1** to G-quadruplexes’ DNA present in the promoter region (c-myc and c-kit), thrombin binding aptamer (TBA) and human telomeric region (a-core and a-coreTT) by UV-Vis spectroscopy, circular dichroism (CD) spectroscopy, thermal melting studies, TRAP assay and FRET-melting assay [23] experiments.

## 2. Results and Discussion

### 2.1. UV-Vis Absorption Titration

**Figure 2:**
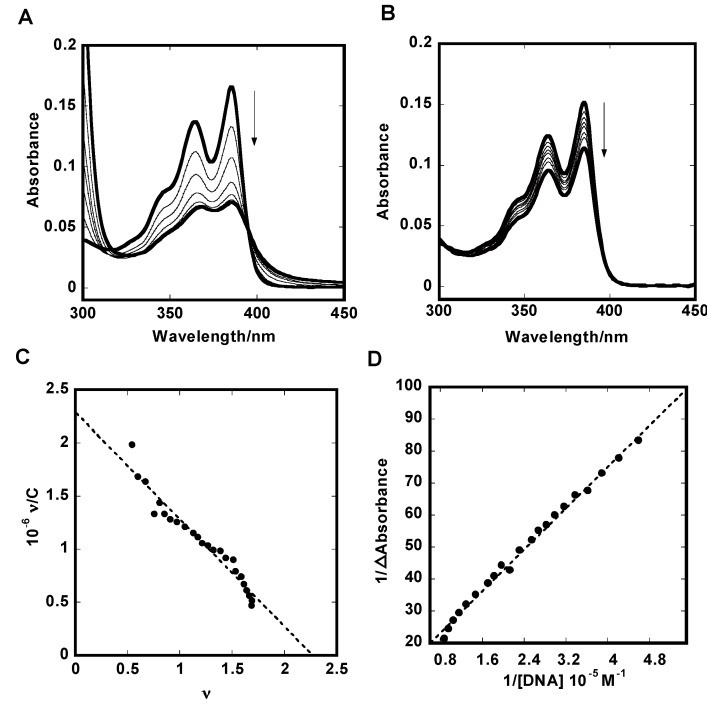
Spectral shifts of 5 μM **1** on titration with 0, 1.4, 2.9, 4.4, 5.8, 8.7 and 14 μM a-core (**A**) or 0, 2.0, 5.0, 10, 20, 30, and 40 μM dsDNA (**B**) in 50 mM Tris-HCl (pH7.4) and 100 mM KCl. Scatchard plots for binding of **1** to a-core (**C**) and Benesi-Hildebrand plot for binding of **1** to dsDNA (**D**).

To obtain the binding constant and the number of bound molecules for the interaction of **1** and non-cyclic naphthalene diimide **2** with different DNA forms such as human telomere (a-core and a-coreTT) [4,24,25], promoter region (c-kit and c-myc) [26,27,28] and thrombin-binding aptamer (TBA) [29,30] their absorption spectra were investigated. Figure 2A shows a representative spectrophotometric titration of **1** with human telomeric G-quadruplex DNA (a-core) in K^+^ ion. It shows a maximum absorption at 384 nm. Addition of increasing amounts of G-quadruplex DNAs to **1** resulted in large hypochromicities (45%–60%) and a noticeable small red shift (3–8 nm) was observed. These spectral features are suggestive of end-staking binding rather than groove binding (Supplementary Figure S1). We observed isosbestic points at 392 nm and 395 nm of **1** for G-quadruplex DNAs and duplex DNA, respectively. The presence of isosbestic points indicated the equilibrium between the bound and free ligand. For comparison, we also investigated the interaction of **1** with dsDNA. Upon the addition of increasing amounts of dsDNA to **1**, smaller hypocromic shifts (25%–30%) and red shifts (2–4 nm) were observed than for G-quadruplexes DNA, suggesting this compound is not a good dsDNA binder (Figure 2B). The Scatchard plot representing the binding between **1** and a-core (KCl) is presented in Figure 2C. The Scatchard plot was analyzed by the McGhee-von Hippel Scatchard equation [31]. The solid line in Figure 2C represents the best fit of the experimental value to the McGhee-von Hippel equation. For dsDNA saturation of binding curves was not achieved; therefore, estimation of *K* values using the Scatchard equation was impossible. However, *nK* values were estimated using the Benesi-Hildebrand method [32]. Ligand binding affinity to dsDNA does not depend on the nature of the metal cation, such as sodium and potassium ions. In the presence of sodium and potassium ions compound **2** binds to dsDNA approximately 20 times stronger than **1**.

The intrinsic binding constants (*K*) of **1** and **2** to G-quadruplexes DNA and dsDNA are summarized in Table 1. We already reported that **3** carrying a benzene moiety as longer linker chain showed higher binding affinity to either G-quadruplexes DNA or dsDNA than **2** [10]. In our present study, we also found similar binding constant trends for G-quadruplexes DNA in the range of 10^6^–10^7^ M^−1^ with *n* = 2, which are an almost five times higher binding affinity of **1** compared with the non-cyclic derivative **2**. Comparing with **3** [10], **1** showed almost three times higher binding affinity to a-core and 70 times weaker binding to dsDNA. Compound **1** showed approximately 200 higher selectivity to G-quadruplexes DNA than the previously reported **3** [10]. The comparison suggests that the shorter cyclic linker chain **1** showed higher specific binding to G-quadruplex DNAs than the previously reported longer linker chain compound **3** [10].

**Table 1:** Binding parameters and melting temperatures of **1** and **2** with a-core, a-coreTT, c-kit, c-myc, TBA and dsDNA.

DNAs	1		2		Tm/°C	∆Tm/°C	
	10^−6^ K/M^−1^	*n*	10^−6^ K/M^−1^	*n*	DNAs	1	2
a-core (K^+^)	10 ± 0.5 ^a^	1.5	1.6 ± 0.2 ^a^	1.4	69 ^c^	15	5
a-core (Na^+^)	1.0 ± 0.04 ^a^	2.2	0.73 ± 0.09 ^a^	1.0	57 ^c^	-	1
a-coreTT (K^+^)	6.1 ± 0.4 ^a^	1.8	1.9 ± 0.3 ^a^	1.3	63.5 ^c^	18	-
TBA (K^+^)	3.5 ± 0.2 ^a^	1.4	0.49 ± 0.04 ^a^	1.1	50.5 ^c^	11	-
c*-*kit (K^+^)	1.9 ± 0.18 ^a^	1.4	0.74 ± 0.05 ^a^	1.7	54 ^d^	11	-
c-myc (K^+^)	4.0 ± 0.3 ^a^	1.7	1.5 ± 0.2 ^a^	1.6	70 ^e^	15	-
dsDNA (K^+^)	0.037 ^b^	-	0.60 ± 0.04 ^a^	2.8	49 ^c^	0.3	12
dsDNA (Na^+^)	0.037 ^b^	-	0.60 ± 0.08 ^a^	3.0	49 ^c^	0.3	12

Condition: Binding constant (*K*): 50 mM Tris-HCl (pH 7.4) and 100 mM NaCl or KCl; ^a^: Scatchard analysis (*K*); ^b^: Bensi-Hildebrand analysis (*nK*); Thermal melting: [ligand]:[DNA] = 2:1, 50 mM Tris-HCl (pH 7.4); ^c^: 100 mM NaCl or KCl; ^d^: 20 mM KCl; ^e^: 5.0 mM KCl.

In comparison with our previous report [10], the linker chain of **1** and **3** may play an important role in the binding with G-quadruplexes DNA over dsDNA. The amide chains of **1** and **3** might be more effective in reducing binding with dsDNA because of the NDI moiety site blocks the aliphatic chain from intercalating in the benzene part. Moreover, the benzene part itself also prevents binding of **1** and **3** from threading intercalations with dsDNA. However, as we have reported earlier [33], **1** and **3** showed affinity to calf thymus DNA (CT-DNA), Poly[d(A-T)]_2_ and Poly[d(G-C)]_2_ due to hydrophobic interaction between cyclic NDI derivatives and dsDNA. Compound **1** showed lower binding affinity to CT-DNA, poly[d(A-T)]_2_ and poly[d(G-C)]_2_ than **3** perhaps because of steric reasons, whereas, according to the computer modeling, the NDI moiety site of cyclic NDI derivatives incorporated to end staking onto the G-quartet. We have observed that **1** showed higher selectivity to G-quartet than **3** because the tertiary amino chain in the linker chain of **1** may have more compatibility to bind specifically with the G-quartet plane than the piperazine linker chain of **3**.

In the presence of sodium ions the binding affinities of both ligands to basket-type tetraplex structures were much lower than G-quadruplexes in potassium solution. We already explained that this might be due to the fact that basket type a-core crosses its oligonucleotide chain over the G-quartet diagonally and disrupts access of **1** to the G-quartet plane [10].

In our present study, all the G-quadruplexes DNA showed higher binding constants (*K*) with **1** than dsDNA. In potassium ion solution, **1** exhibited the highest binding affinity for mixed hybrid type a-core [24] with *K* = 1 × 10^7^ M^−1^, whereas diminished the binding affinity to dsDNA with *nK* = 3.7 × 10^4^ M^−1^. The binding data indicated that **1** has a 270-fold preference for a-core over dsDNA. Table 1 shows the binding constants (*K*) are *K* = 6.1 × 10^6^ M^−1^ for a-coreTT (hybrid type-2) [25], *K* = 1.9 × 10^6^ M^−1^ for c-kit (parallel propeller type) [27], *K* = 4.0 × 10^6^ M^−1^ for c-myc (parallel type) [27], *K* = 3.5 × 10^6^ M^−1^ for TBA (antiparallel chair type) [30] with **1**, which represent a 165-, 51-, 108- and 95-fold binding preference over dsDNA (*K* = 3.7 × 10^4^ M^−1^), respectively. The ratio of ligand per dsDNA used for binding was *n* = 3, a reasonable result considering that a typical intercalator covers two base pairs upon binding to dsDNA, in addition to the expected relative difficulty in binding at terminal sites. The binding number of a ligand with G-quadruplexes DNA was estimated to be *n* = 2, which may agree with an end-stacking binding of **1** to the external G-quartet planes of quadruplexes.

According to the above result, new compound **1** showed G-quadruplexes DNA structure-specific binding. Compound **1** showed the highest affinity to a-core DNA, which exhibits mixed type hybrid (hybrid-1 and hybrid-2) structures in K^+^ [24] and possessed more than two drugs staking plane and binding loops. Computer modeling showed that **1** was stacked and bound to a mixed hybrid structure at various G-quadruplex staking planes, whereas a-coreTT exhibited hybrid-2 [25] type structures which have two G-tetrads staking planes and two binding sites. For this reason we observed that **1** showed the highest affinity with a-core. TBA exhibited an antiparallel chair type [29,30] structure which has G-quartet staking planes and binding loops, while c-kit and c-myc exhibited parallel type [27] propeller structures which possess two G-quartet staking planes, but binding loops are unusual for **1**. According to the binding data, new compound **1** was revealed to be a most preferable and specific binder to telomeric G-quadruplexes DNA than promoter regions’ G-quadruplex DNA as well as thrombin binding aptamer. The binding studies results are consistent with thermal melting studies where a-core showed the highest stabilization with **1**. The binding site size (*n* values) obtained from binding studies of G-quadruplexes and **1** are consistent with the Job plot analysis from CD studies (Supporting Information Figure S2).

### 2.2. Circular Dichroism (CD) Studies

**Figure 3:**
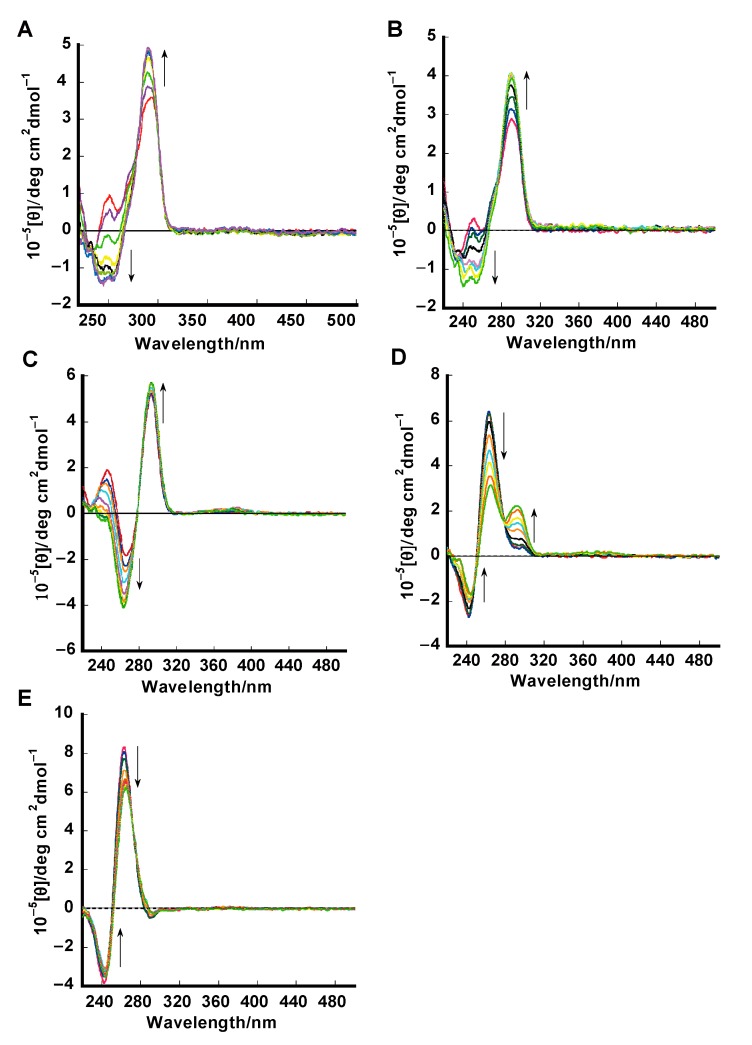
CD spectra of 1.5 μM of a-core (**A**); a-coreTT (**B**); TBA (**C**); c-kit (**D**); c-myc (**E**) in 50 mM Tris-HCl (pH 7.4), 100 mM KCl in addition of **1** (0, 0.38, 0.75, 0.80, 2.25, and 3.00 μM) at 25 °C.

CD is a powerful method to differentiate the parallel, anti-parallel, and mixed-type secondary structure of G-quadruplex DNA. Compound **1** was interacted to investigate the effect of the compound binding on the conformation of the G-quadruplexes, which is shown in Figure 3. The CD spectrum of human telomere (a-core) G-quadruplex DNA showed a negative peak at 240 nm, a shoulder peak at 265 nm and a positive peak at 290 nm (Figure 3A) in buffer containing 100 mM KCl, supportive of a mixed hybrid type (hybrid-1 and hybrid-2) G-quadruplex structure [34,35]. The small positive peak at 265 nm was transformed increasingly into a negative peak at 260 nm together with an increase of the positive peak at 290 nm upon the addition of **1**, suggesting the induction of a hybrid type structure. After addition of **1**, a-core structure conformation changed a little from mixed hybrid type to hybrid-1 type [36]. In our previous study, we reported that in the presence of Na^+^ ions, telomeric DNA exists in an antiparallel basket-type conformation. Upon addition of **1**, this antiparallel basket-type structure was also retained [10].

The CD spectrum of human telomere (a-coreTT) G-quadruplex in buffer containing 100 mM KCl exhibits a negative peak at 240 nm, a shoulder peak at 265 nm and a positive peak at 290 nm (Figure 3B) supportive of a hybrid-2 type G-quadruplex structure [34,35]. The small positive peak at 265 nm is transformed increasingly into a negative peak at 260 nm together with an increase of the positive peak at 290 nm upon the addition of **1**, suggesting the induction of a hybrid type structure. After the addition of **1**, a-core structure conformation changed a little from hybrid-2 type to hybrid-1 type [36].

In the presence of 100 mM KCl thrombin-binding aptamer (TBA, Figure 3C) exhibited a positive peak at 290 nm, and a negative band at 250 nm, supportive of an anti-parallel chair type G-quadruplex structure [30,37]. Upon the addition of **1**, the negative peak transformed increasingly into at 260 nm together with an increase of the positive peak at 290 nm, suggesting that the binding of **1** apparently does not disturb the structure of TBA, which is consistent with previous reports [37].

Both c-myc and c-kit G-quadruplex (Figure 3D,E) exist in the presence of K^+^ ions as a parallel structure, which has a characteristic positive peak centered around 262 nm, a negative peak at 241 nm and a small shoulder peak at 290 nm [38,39]. After the addition of **1** to c-myc and c-kit G-quadruplex, a decrease of the CD peaks at 241 nm and 262 nm was observed, with no other significant change in the spectrum and the parallel structure was not change, which suggests ligand-dependent disruption of staking of G-quadruplex DNA. Upon the addition of **1**, the c-kit structure shoulder peak at 290 nm increased little. This effect has also been observed previously by many research groups [40].

The Job plot analysis by CD studies (Supporting Information Figure S2) showed that CD studies of G-quadruplexes and **1** are consistent with binding studies where similar binding site sizes (*n* values) are obtained.

### 2.3. Thermal Melting Studies

**Figure 4:**
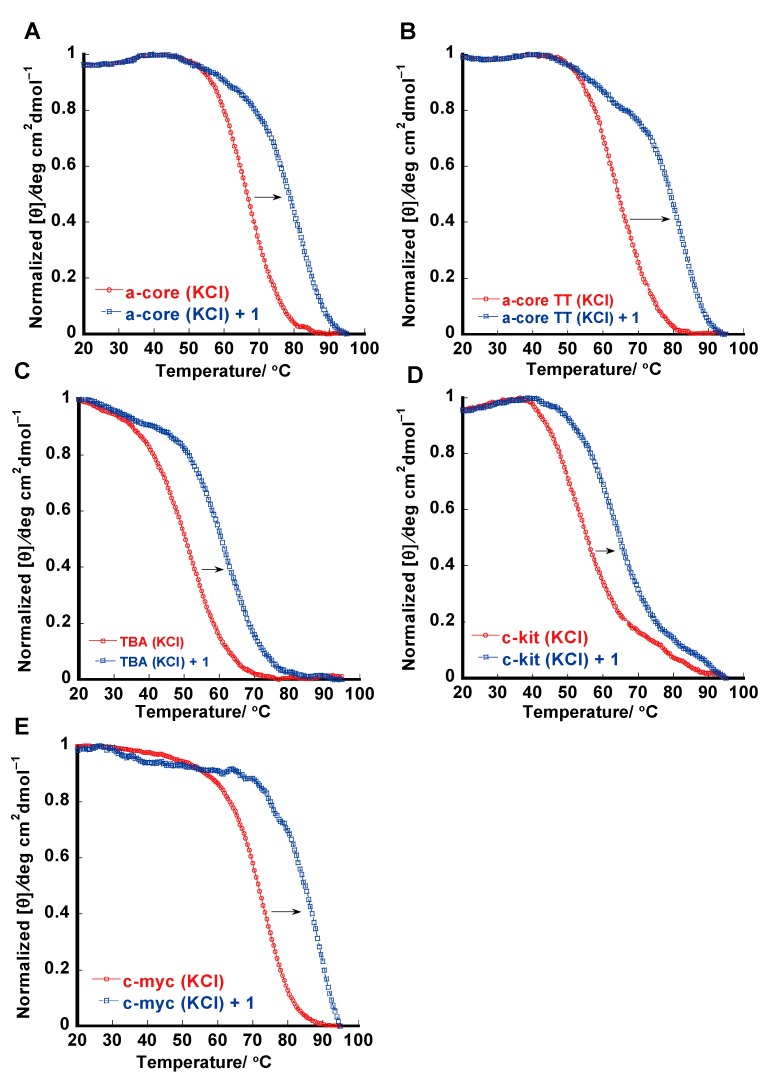
Melting profiles for a-core (**A**); a-coreTT (**B**); TBA (**C**); c-kit (**D**); c-myc (**E**) in the absence or presence of **1** in 50 mM Tris-HCl (pH 7.4); 100 mM KCl (**A**–**C**); 20 mM KCl (**D**); 5.0 mM KCl (**E**) and [ligand]:[DNA] = 2:1.

Thermal stabilization of various G-quadruplexes DNA and dsDNA in the presence of **1** was studied using the CD melting and UV-Vis melting experiment (Figure 4). Thermal melting of hybrid type telomeric quadruplex DNA (a-core and a-coreTT) was monitored at 290 nm in the presence of K^+^ [25].

The T_m_ value was observed around 69 °C for a-core without **1** (Figure 4A). We observed that the interaction of **1** with telomeric DNA quadruplex enhanced the stability by 15 °C for a-core and 18 °C for a-coreTT, which was approximately 3 °C higher for a-coreTT than a-core G-quadruplexes DNA (Table 1). Researchers already reported that thermal melting increased after addition of base to oligonucleotides [41]. In our previous report [10], we observed from the absorption spectra that **3** had the lowest binding affinity to a-core in sodium ion solution. Furthermore, dsDNA was monitored by UV-Vis melting studies. After the addition of a 1-fold **1** concentration, only a slight increase (up to 0.3 °C) in thermal stability was observed (Table 1 and Supplementary Figure S3). These results underscore the fact that **1** selectively stabilizes telomeric quadruplex DNA as well as promoter and thrombin binding aptamer G-quadruplex DNA over dsDNA. In comparison with our previous report [10], compound **1** showed high stabilizing effect to a-core and very weak stabilizing effect to ds DNA.

The melting temperatures of antiparallel chair type thrombin binding aptamer quadruplex DNAs (TBA) were monitored at 290 nm. Compound **1** increased the T_m_ of TBA by 11 °C (Table 1). These results are consistent with previously published articles [30,37]. The melting temperatures of parallel promoter quadruplex DNAs such as c-kit and c-myc were monitored at 263 nm [39,40]. In the case of the highly stable parallel c-kit and c-myc quadruplex DNA was highly stable at high salt concentration and a stable baseline curve was not achieved even above 90 °C, so it is not possible to measure an accurate T_m_ in this case, so we measured T_m_ for c-kit and c-myc at low salt concentration. Compound **1** increased the T_m_ of c-kit by >11 °C at 20 mM K^+^ ion and the T_m_ of c-myc by >15 °C at 5 mM K^+^ ion solution (Table 1). This type of performance of c-kit and c-myc is consistent with previously published articles [39,40]. According to the above result, we can conclude that **1** preferably stabilizes telomeric quadruplex DNA than promoter and thrombin binding aptamer G-quadruplex DNA. CD melting results are consistent with binding and competition assay studies, where **1** showed preferable binding to human telomeric G-quadruplex.

### 2.4. TRAP Assay

**Figure 5:**
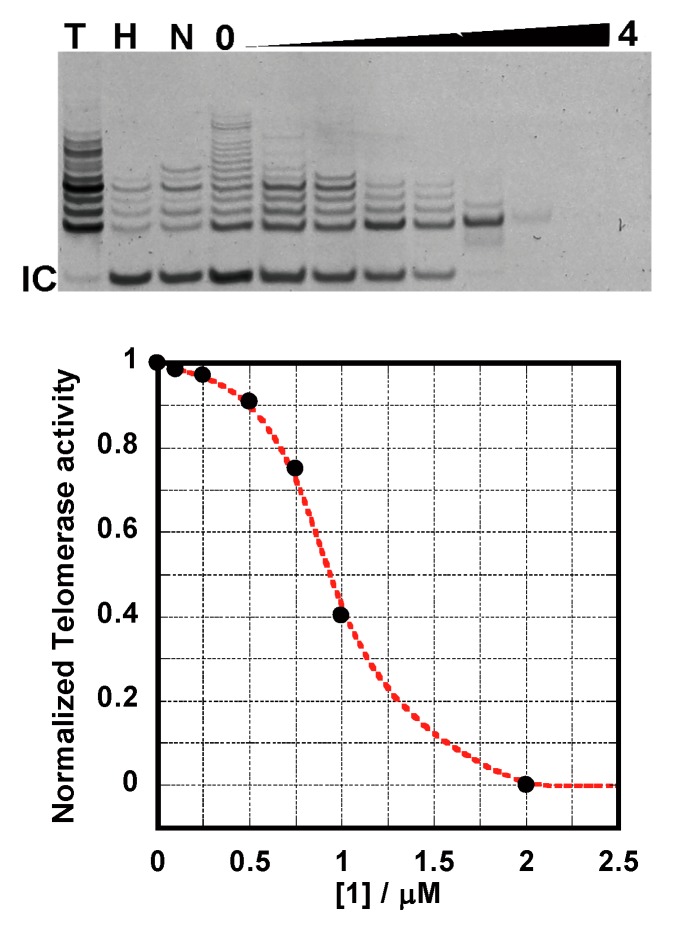
Telomerase inhibition by **1**. The gel shows the effect of increasing concentrations of **1** (0, 0.1, 0.25, 0.5, 0.75, 1.0, 2.0, 3.0, 4.0 μM) on telomerase activity. Concentrations of 2.0–4.0 μM **1** lead to the disappearance of all PCR products. IC_50_s were determined as follows: ligand concentration under half telomerase activity with no ligand.

Once the G-quadruplexes DNA stabilization was established for **1**, it was important to test whether the molecule inhibits telomerase activity. To evaluate the abilities of these compounds to inhibit telomerase, the telomeric repeat amplification protocol (TRAP assay) [10,22] was carried out using various amounts of **1** (Figure 5). The assay clearly shows that **1** is a potent inhibitor of telomerase with activity in the submicromolar range (IC_50_) 0.9 μM. This result suggests that the TS-primer extends the length to form a tetraplex structure and **1** binds to it and stabilizes its structure to inhibit the telomerase reaction. The values obtained from the TRAP assay are comparable to those of previously reported derivatives [10,22]. A number of small ligands have been discovered to inhibit the function of telomerase by stabilizing G-quadruplexes DNA structures [7]. The excellent IC_50_ for telomerase inhibition by **1** (0.9 μM) comes from its binding constant (*K* > 10^7^ M^−1^). It is suggested that this macrocyclic compound **1** may deserve biological assays with cancer cell lines to represent a suitable candidate drug target to DNA quadruplexes.

### 2.5. FRET-Melting Assay

**Figure 6:**
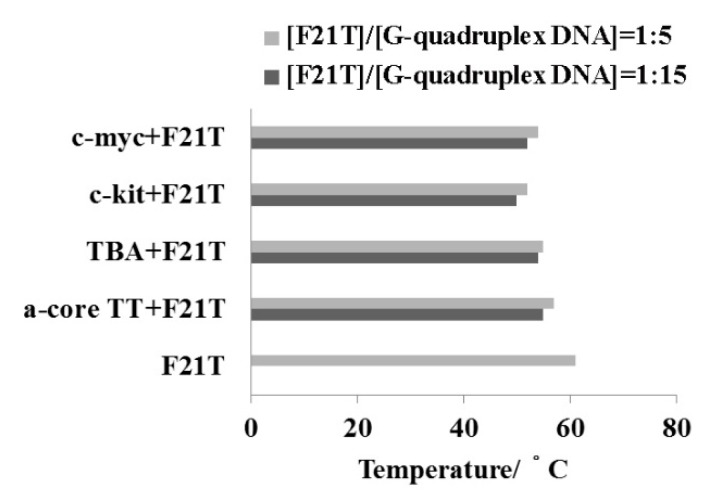
FRET-melting assay of human telomeric DNA (a-coreTT) (1.0 or 3.0 μM), promoter region’s DNA (c-kit & c-myc) (1.0 or 3.0 μM) and thrombin-binding aptamer (TBA) (1.0 or 3.0 μM) with F21T (0.2 μM) in the presence of **1** (0.4 μM). Experiments were performed at 25 °C in 100 mM Tris-HCl buffer pH 7.4 containing 150 mM KCl.

The sequence and structural selectivity of different DNA binding agents has been previously explored by use of a thermodynamically rigorous competition assay procedure introduced by Ren and Chaires [23,42]. In this method, different nucleic acid structures are assayed against a common ligand solution. This is a simple method to evaluate specificity toward quadruplexes [43]. It has been already reported that F21T showed a Tm value around 50 °C [23] that increased by 11 °C after incorporation of **1** with F21T. Comparison with other G-quadruplexes DNA is shown in Figure 6. Compound **1** displays a strong preference for binding to F21T quadruplex structure which corresponds to the human telomeric G-rich motif than other quadruplexes DNA structures.

### 2.6. Computer Modeling

**Figure 7:**
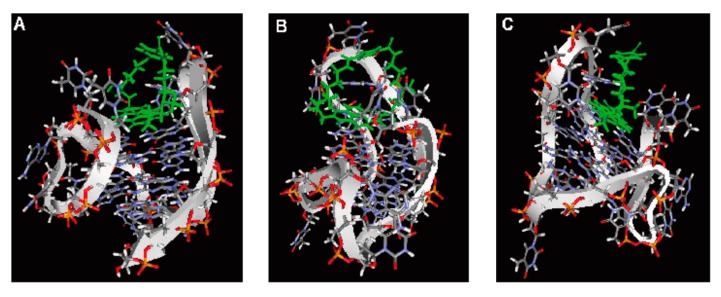
Computer modeling of **1** interaction with mixed hybrid G-quadruplex DNA structure (**A**–**C**).

The computer-modeling structures consisting of **1** with mixed hybrid types G-quadruplex DNA (a-core) are shown in Figure 7. In this article we proposed a model involving an end staking binding mode between **1** and mixed hybrid types G-quadruplex DNA (a-core), which are consistent with our previously published article [22]. The computer modeling showed that the **1** molecule stacked and bound to different G-quartet plane of mixed hybrid types G-quadruplex DNA (a-core).

## 3. Experimental Section

### 3.1. Materials

The seven G-rich oligonucleotides: a-core (5′-AGGG(TTAGGG)_3_-3′), a-coreTT (5′-AGGG (TTAGGG)_3_TT-3′), TBA (5′-GGTTGGTGTGGTTGG-3′), c-kit (5′-AGGGAGGGCGCTGGGAG GAGGAGGG-3′), c-myc (5′- TGAGGGTGGGGAGGGTGGGGAA-3′) and dsDNA composed of two complementary strands: (5′-GGGAGGTTTCGC-3′) and (5′-GCGAAACCTCCC-3′) were purchased from Genenet Co. (Fukuoka, Japan) and used without further purification. The following extinction coefficients were used for quantification of nucleic acid solutions (unit of ε was M^−1^ cm^−1^): 114,000 for 5′-GGGAGGTTTCGC-3′; 108,600 for 5′-GCGAAACCTCCC-3′; 228,500 for a-core (5′-AGGG (TTAGGG)_3_-3′); 245,100 for a-coreTT (5′-AGGG(TTAGGG)_3_TT-3′); 143,300 for TBA (5′-GGTTG GTGTGGTTGG-3′); 260,100 for c-kit (5′-AGGGAGGGCGCTGGGAGGAGGAGGG-3′); 229,900 for c-myc (5′-TGAGGGTGGGGAGGGTGGGGAA-3′). Before use, oligonucleotide solutions in 50 mM Tris-HCl buffer (pH 7.4) containing 100 mM NaCl or KCl were heated to 95 °C and annealed by slowly cooling to room temperature. Guanine-rich telomere oligonucleotide sequence (5′-d-GGGTTAGGGTTAGGGTTAGGG3′), dual label with FAM (fluorescent donor) and TAMRA (fluorescent acceptor) at the 5′ and 3′ end, called ‘F21T’, respectively, were purchased from Sigma-Aldrich (St. Louis, MO, USA). The synthesis procedure of **1** was described in detail in a previous article [33]. Compound **2** was synthesized as described previously [44]. The 2.0 M KCl, and 5.0 M NaCl aqueous solutions were obtained from Life Technologies (Carlsbad, CA, USA). 1.0 M Tris-HCl (pH 7.4) buffer was obtained from Sigma-Aldrich. GoTaq Hot Start polymerase was purchased from Promega (Madison, WI, USA). TRAPese kit was obtained from EMD Millipore (Billerica, MA, USA).

### 3.2. UV-Vis Titration Experiments

Absorption spectra were measured on a U-3310 spectrophotometer (Hitachi, Tokyo, Japan) with a 1 cm path-length quartz cell and were recorded in the 200–600 nm range at 25 °C. UV-Vis absorption titrations were carried out by the stepwise addition of 200 μM/strand of G-quadruplexes DNA or dsDNA solution to a UV-cell containing 5.0 μM solutions of **1** or **2**. The measurements were performed in a 50 mM Tris-HCl buffer (pH 7.4) containing 100 mM NaCl or KCl. Binding data obtained from spectrophotometric titration of increasing concentrations of drug to a fixed concentration of DNA was cast into the form of a Scatchard plot of *ν*/*C* against *ν*. The Scatchard plot was analyzed by the Scatchard equation: *ν*/*C* = *K*(*n*–*ν*) [31], where *ν* is the stoichiometry (the number of ligand molecules bound per moles of base pair), C is the free ligand concentration, *K* is the observed binding constant, and *n* is the number of base pairs excluded by the binding of a single ligand molecule. For duplex oligonucleotides saturation of binding curves was not achieved, so *K* values were estimated using Benesi-Hildebrand method 1/ΔAbs = 1/(*l*Δε [ligand]) + 1/(*nKl*Δε [ligand]) × (1/DNA) [32] with the assumption that the ligand/oligonucleotide complex with 1:1 stoichiometry is formed (Table 1), where Δε is a molar absorptivity change of ligand and *l* is 1 cm. Scatchard plots were prepared using absorption changes at the specific wavelength 383 nm upon the addition of various concentrations of dsDNA. Scatchard plots were prepared using the data in a range of approximately 30%–80% bound region of **1** and dsDNA. The binding data were analyzed with KaleidaGraph software, using the Levenberg-Marquardt algorithm to determine parameters *K*_b_ and *n*.

### 3.3. Circular Dichroism (CD) Spectral Measurements

Various concentrations (5.0 to 50 μM) of **1** or **2** were added to 1.5 μM/base pair G-quadruplexes DNA in a 50 mM Tris-HCl buffer (pH 7.4) containing 100 mM NaCl or KCl at 25 °C, and CD spectra taken at a scan rate 50 nm/min on a J-820 spectropolarimeter (Jasco, Tokyo, Japan). Other conditions were: response 2 s, data interval 0.1 nm, sensitivity 100 mdeg, band width 2 nm, and scan number 4 times.

### 3.4. Thermal Melting Experiments

Melting curves of G-quadruplexes DNA or dsDNA were measured on a Hitachi 3300 spectrophotometer (heating rate of 0.5 °C/min to 90 °C) or Jasco J-820 spectrophotometer (response, 100 mdeg; response, 8 s; data collecting interval, 0.5 °C; bandwidth, 2 nm) equipped with a temperature controller, respectively. The melting curves based on circular dichroism (CD) at 290 nm of a-core, a-coreTT, and TBA, 262 nm of c-kit and c-myc or 260 nm of dsDNA were measured in 50 mM Tris-HCl (pH 7.4) containing 100 mM NaCl or KCl (for c-kit 20 mM KCl and for c-myc 5 mM KCl). A mixture of 1.5 μM a-core, a-coreTT, TBA, c-kit, c-myc, dsDNA and 3.0 μM of **1** or **2** was placed in a cell of 1 cm in light path length (total 3 mL). Ligand-DNA ratio was set at 2:1.

### 3.5. TRAP Assay Experiments

Telomeric repeat amplification protocol (TRAP) assay was performed using published procedure [10,22]. TRAPeze Telomerase Detection Kit from EMD Millipore was used. Briefly, TS forward primer was elongated by telomerase in TRAP buffer (20 mM Tris-HCl pH 8.3, 1.5 mM MgCl_2_, 63 mM KCl, 0.05% Tween 20, 1.0 mM EGTA) containing 0.05 mM dNTPs, 0.4 μM TS primer, 0.4 μM primer Mixed (RP primer, K1 primer, TSK1 primer) and 2.0 units of GoTaq Hot Start polymerase. The mixture was added to freshly prepared **1** solution from 0.1 to 4.0 μM (0, 0.1, 0.25, 0.5, 0.75, 1.0, 2.0, 3.0, 4.0 μM) and a positive control containing no ligand. Firstly, the elongation step was carried out for 60 min at 30 °C and it was followed by 5 min incubation at 95 °C. Secondly, 35 cycles of PCR were performed (94 °C, 30 s; 62 °C, 1 min; 72 °C, 1 min). Telomerase extension products were analyzed on a denaturing 12.5% polyacrylamide vertical gel prepared in 5 × TBE buffer (89 mM Tris base, 89 mM borate, and 1 mM EDTA, pH 8.0). The electrophoresis was run in 0.7 × TBE buffer for 2 h at 200 V. After electrophoresis gel was stained in 1 × GelStar Nucleic Acid Stain (Takara Bio, Shiga, Japan) in 1× TBE buffer for 30 min and photographed.

### 3.6. FRET-Melting Assay

Fluorescence-based melting competition assays was performed using a previously published procedure [23]. In more recent experiments, a real-time PCR apparatus (MX3000P, Stratagene, La Jolla, CA, USA; or Sigma-Aldrich SYBR Green or DNA engine Opticon, MJ Research, Waltham, MA, USA) is used, allowing the simultaneous recording of 32–96 independent samples as first proposed by S. Neidle and co-workers [23]. The protocol used for our experiments is the following: a first step of equilibration at the lowest temperature (5 min at 25 °C) and a stepwise increase of 1 °C every minute for 72 cycles to reach 95 °C. The buffer 100 mM Tris-HCl (pH 7.4) containing 150 mM NaCl or KCl and 0.4 μM **1** was used for all experiments. The thermal denaturation profile of the oligonucleotide F21T (0.2 μM) and G-quadruplexes DNA (a-coreTT, TBA, c-kit and c-myc) (1.0 or 3.0 μM) were measured in the presence of **1** (0.4 μM). The ratio of F21T and G-quadruplexes DNA was used 1:5 or 1:15 at 0.4 μM **1**. Fluorescence-based melting assays competition measurements were performed with F21T dual label with FAM (fluorescent donor) and TAMRA (fluorescent acceptor) at the 5′ and 3′ end from Sigma-Aldrich at a heating rate of 1 °C/min. The recording is performed after 1 min stabilization. Typically three replicate experiments were performed, and average values are reported. Finally, the amount of ligand bound to the DNA was quantified by fluorescence after the digestion of the oligonucleotide (λ_ex_ and λ_em_ were set to 490 and 520 nm for oligonucleotides, respectively).

### 3.7. Computer Modeling

Molecular modeling of these complexes was constructed by MOE 2011.10 (http://www.chemcomp.com/). Compound **1** was placed on the binding site of mixed hybrid types G-quadruplex DNA (a-core) and energy minimization of these complexes was carried out. The molecular dynamics calculation of these mineralized complexes was further carried out until **1** was located in the binding site as stable condition. Finally, energy minimization of the complexes was obtained as shown in Figure 7. These calculations were used the force field of MMFF94x.

## 4. Conclusions

We have a synthesized new type of ligand **1**, carrying a benzene moiety as linker chain and studied its interaction with different types of G-quadruplexes DNA. We have compared this study with our previously reported **3** [10] which has a long linker chain than **1**. Compound **1** exhibited high binding affinity in the range of 10^6^–10^7^ M^−1^ to G-quadruplexes DNA and reduced binding affinity to dsDNA. The binding data (Table 1) indicated that **1** has 270-fold preferential binding for a-core, 165-fold for a-coreTT, 51-fold for c-kit, 108-fold for c-myc, 95-fold for TBA over dsDNA. The binding stoichiometry of **1** for G-quadruplex is 2:1, suggesting a staking binding mode. Compound **1** revealed 200-fold higher binding selectivity compared with our previously reported **3** [10]. We have observed that **1** revealed preferable binding to mixed hybrid types structure of telomeric G-quadruplex DNA (a-core) over parallel types of promoter region’s G-quadruplex DNA (c-kit and c-myc) and antiparallel chair types of thrombin binding aptamer (TBA). The CD spectra showed that **1** stabilized G-quadruplexes DNA structure. Upon the addition of **1** to a-core the CD spectra showed little change indicating a mixed hybrid structure and little changed to the hybrid-1 type G-quadruplex structure [36]. Thermal melting measurements indicated that **1** highly stabilized the G-quadruplexes DNA structure. Compared with our previous report [10], **1** increased ∆T_m_ by 5–8 °C. We have performed competitive assays in order to determine the binding selectivity among the G-quadruplexes DNA, and **1** showed highly preferable stabilization of human telomeric G-quadruplex sequence (F21T). This novel compound **1** can also inhibit the telomerase activity at low submicromolar concentration. These results indicated that **1** is an important class of G-quadruplex stabilizing ligand compared with dsDNA.

## Supplementary Materials

Supplementary data (UV-Vis absorption spectra, Job plot analysis of CD spectra, UV-Vis melting curves of dsDNA and competition assay curves) associated with this article can be found, in the online version, at http://www.mdpi.com/1420-3049/20/06/10963/s1.
