# Supplementary material for: A Selective G-Quadruplex DNA-Stabilizing Ligand Based on a Cyclic Naphthalene Diimide Derivative
Source: Molecules. 2015 Jun 12;20(6):10963–79. doi: 10.3390/molecules200610963 (PMC6272171; doi:10.3390/molecules200610963)
Supplement: Supplementary file 1 [file molecules-20-10963-s001.pdf]

Supporting Information

List of Figures Pages

Figure S1.	UV-Vis absorption spectra of **1** with G-quadruplexes and dsDNA.	S2
Figure S2.	Analysis of CD spectra of **1** and G-quadruplexes DNA interaction by job plot.	S3
Figure S3.	UV-Vis melting profiles for dsDNA (KCl) and dsDNA (NaCl).	S4
Figure S4.	FRET-melting assay of G-quadruplexes DNA with F21T in the presence of **1**.	S4

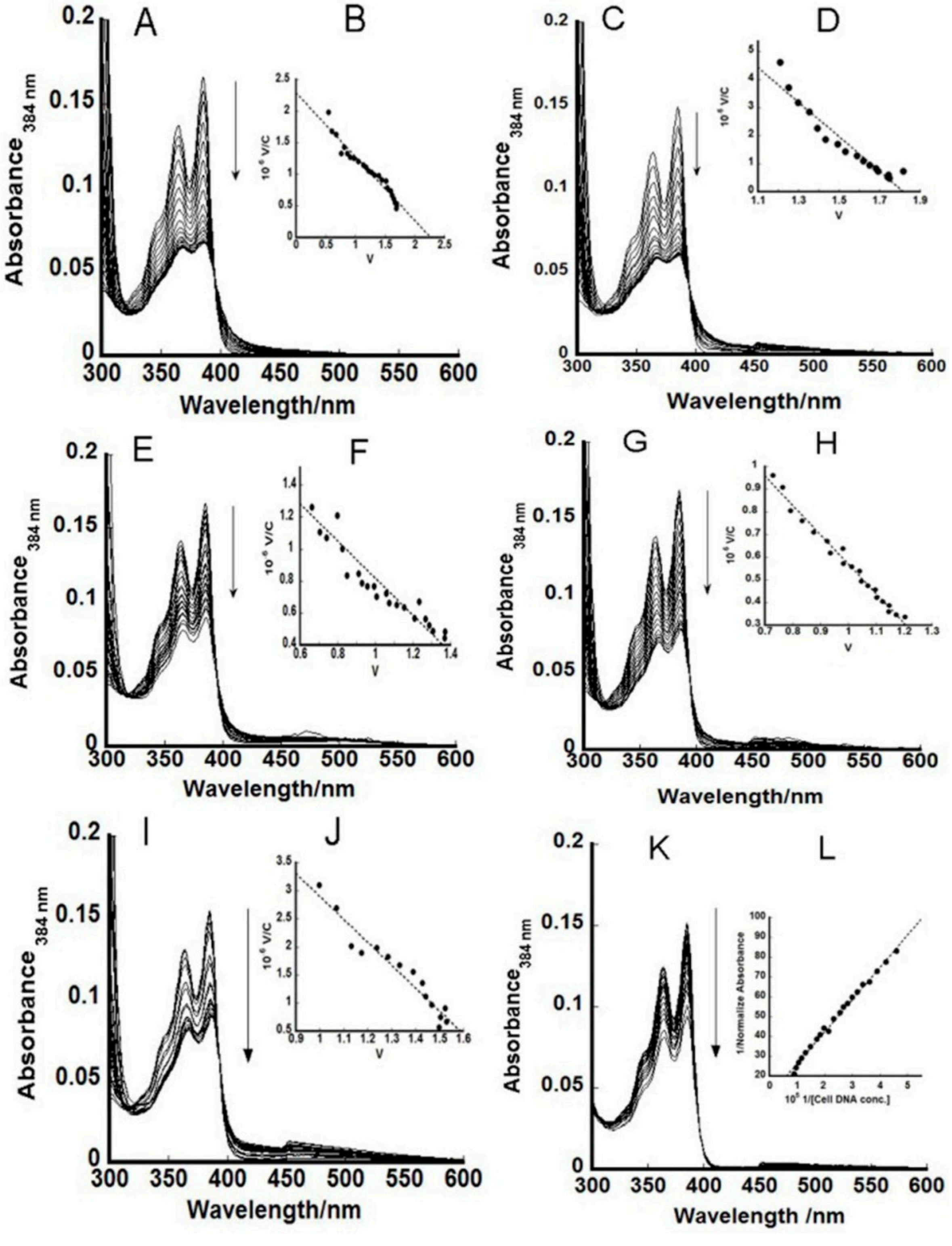

Figure S1. UV-Vis absorption spectra of 5.0 μM **1** in the absence and presence of a-core (**A**); a-coreTT (**C**); TBA (**E**); c-kit (**G**); c-myc (**I**) and dsDNA (**K**) with 0.5, 1.0, 1.5, 2.0, 2.5, 3.0, 4.0, 5.0, 10 and 20 μM respectively. Binding affinities were estimated using the scatchard plot of **1** with a-core (**B**); a-coreTT (**D**); TBA (**F**); c-kit (**H**), c-myc (**J**) and Benesi-Hildebrand plot of **1** with dsDNA (**L**). Experiments were performed at 25 °C in 50 mM Tris-HCl (pH 7.4), 100 mM KCl.

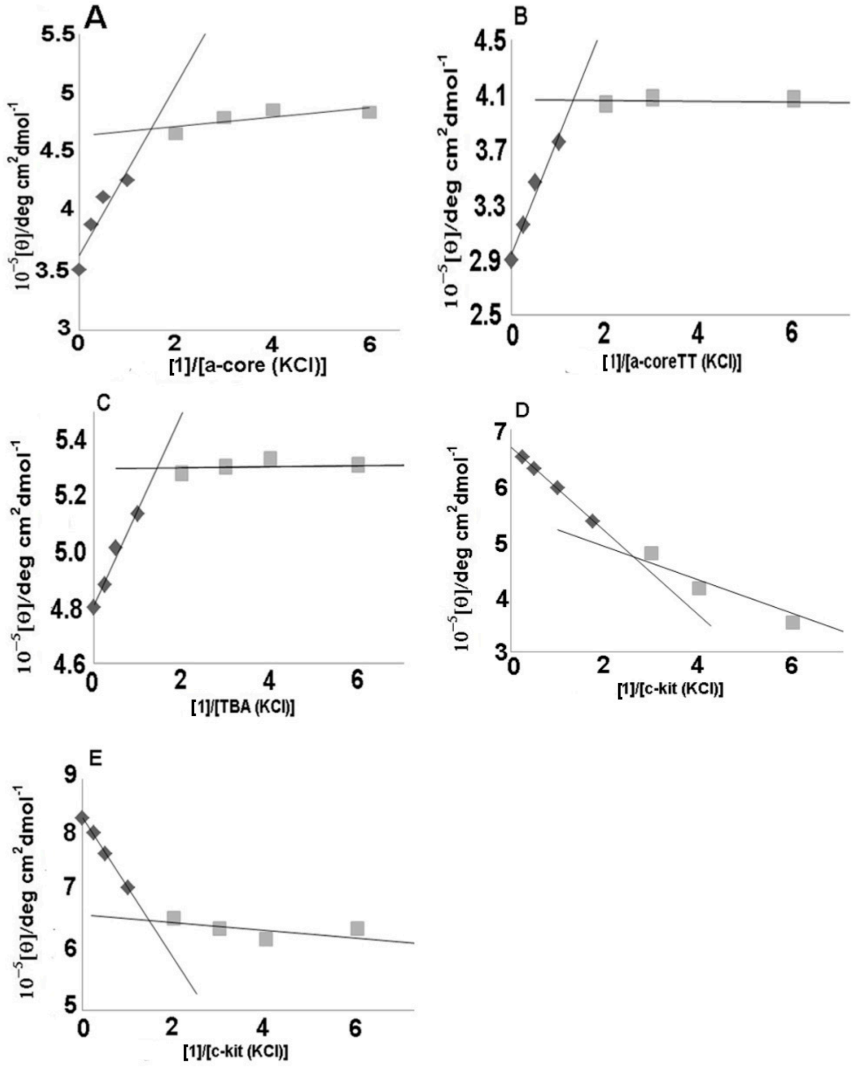

Figure S2. Analysis (by Job plot) of CD spectra of **1** with a-core (**A**); a-coreTT (**B**); TBA (**C**), c-kit (**D**); c-myc (**E**). Molar ellipticity values at 290 nm (a-core, a-coreTT, and TBA) and 263 nm (c-kit and c-myc) were plotted against increased ligand-DNA molar ratio. Experiments were performed at 25 °C in 50 mM Tris-HCl buffer pH 7.4 containing 100 mM KCl. The intersection of data indicated the binding stoichiometry of **1** and G-quadruplexes DNA [1]. We have observed binding stoichiometry n = 1.7 for a-core (**A**); n = 1.5 for a-coreTT (**B**); n = 1.8 for TBA (**C**); n = 2.5 for c-kit (**D**); n = 1.7 for c-myc (**E**). These results are consistent with the stoichiometry from UV-Vis binding studies.

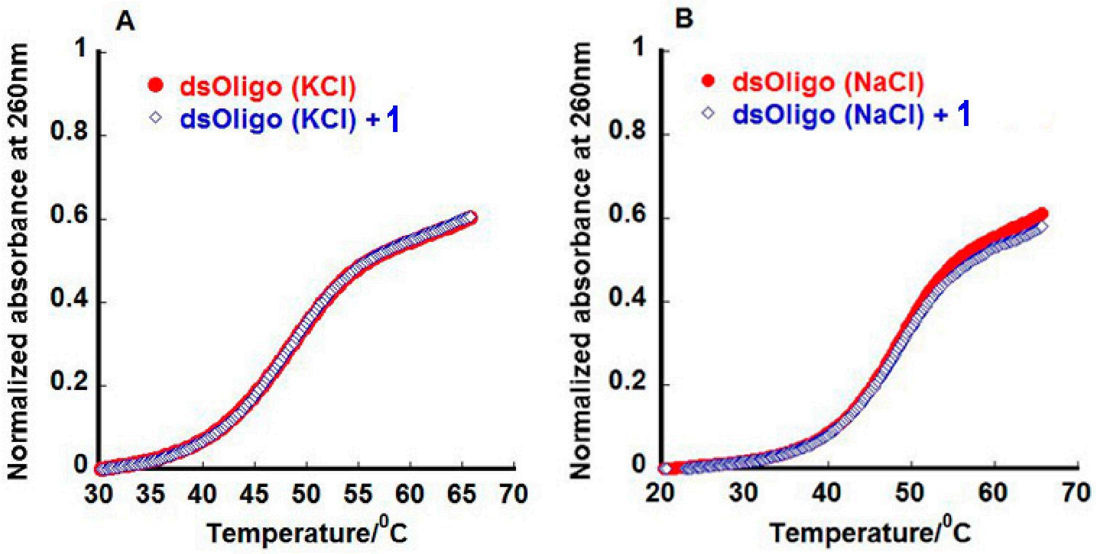

Figure S3. UV-Vis melting profiles for dsDNA (KCl) (**A**) and dsDNA (NaCl) (**B**) in the absence or presence of **1** in 50 mM Tris-HCl (pH 7.4) and 100 mM KCl or 100 mM NaCl, [ligand]:[DNA] = 2:1.

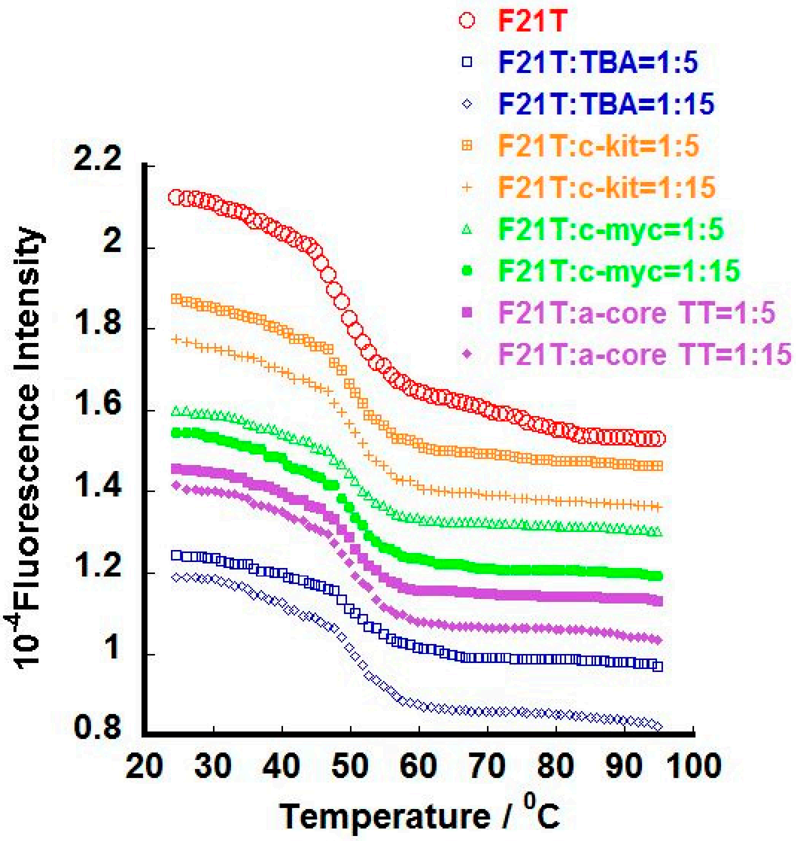

Figure S4. FRET-melting assay of human telomeric DNA (a-coreTT), promoter region's G-quadruplex (c-kit & c-myc) and thrombin-binding aptamer (TBA) with F21T (0.2 μM) in the presence of **1** (0.4 μM). Experiments were performed in 100 mM Tris-HCl buffer (pH 7.4) containing 150 mM KCl.

Reference

1. Kieltyka, R.; Englebienne, P.; Miotessier, N.; Sleiman, H. Quantifying interactions between G-quadruplex DNA and tranition-metal complexes. In *G-quadruplex DNA Methods and Protocols*, 1st ed.; Baumann, P., Ed.; Humana Press: New York, NY, USA, 2010; pp. 221–256.
